# Aeroembolism in left atrium during catheter ablation of atrial fibrillation in a patient with dextrocardia: a case report and review of the literature

**DOI:** 10.1186/s12872-017-0581-7

**Published:** 2017-06-02

**Authors:** Yun-fan Wang, Xue-jiang Cen, Jian-wu Yu, Bai-ming Qu, Li-hong Wang

**Affiliations:** Department of Cardiology, Zhejiang Provincial Peoples’ Hospital, Shangtang Road 158#, Hangzhou, Zhejiang China

**Keywords:** Aeroembolism, Atrial fibrillation, Catheter ablation, Dextrocardia, Case report

## Abstract

**Background:**

Air embolus penetrating into heart chamber as a complication during percutaneous radiofrequency catheter ablation has been infrequently reported.

**Case presentation:**

A 55-year-old man with dextrocardia who suffered from abdominal pain was suspected to have multiple arterial thromboembolisms, which might have originated from left atrium thrombosis since he had atrial fibrillation. He received oral anticoagulant therapy and catheter ablation of the arrhythmia. During the ablation procedure, an iatrogenic aeroembolism penetrated into the left atrium due to improper operation. Ultimately, the entire air embolus was extracted from the patient, who was free of any aeroembolism events thereafter.

**Conclusions:**

It is essential for an operator to pay full attention to all details of the procedure to avoid an aeroembolism during catheter ablation. In case of aeroembolism, removal by aspiration is an optimal and effective treatment.

**Electronic supplementary material:**

The online version of this article (doi:10.1186/s12872-017-0581-7) contains supplementary material, which is available to authorized users.

## Background

Catheter ablation has been recommended as the first-line therapy for atrial fibrillation (AF) in some select patients [1]. The procedure is usually safe but still not free of complications; they mainly include pulmonary vein stenosis, thromboembolism, atrioesophageal fistula, and post-ablation atrial flutter [2]. During catheter ablation, embolus due to air entry rarely occurs but it is potentially life-threatening. The air embolus could potentially cause acute blood flow interruption to some vital organs, including acute ischaemic stroke, and acute myocardial infarction, and may be fatal in some cases. Here, we present a male patient with dextrocardia and paroxysmal AF who suffered the rare complication of aeroembolism in the left atrium during catheter ablation. However, since the air embolus accumulated in the left atrium, it was successfully aspirated and arterial aeroembolism did not occur. We perform this case report according to the CARE guideline and its methodology.

## Case presentation

**Fig. 1:**
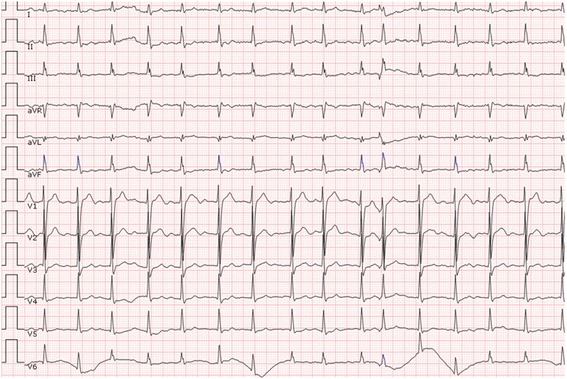
The 12-lead electrocardiography of the patient at admission

**Fig. 2:**
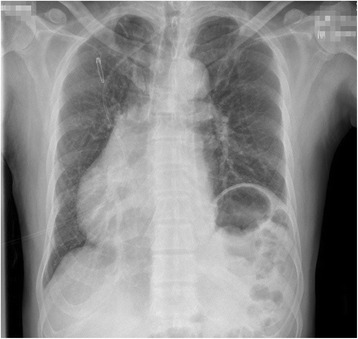
The chest fluoroscopy demonstrated dextrocardia

A 55-year-old man with paroxysmal AF was admitted to the emergency room due to an acute right inferior abdominal pain. He did not present with fever, vomiting, odynuria, abdominal distention, or diarrhoea. His blood pressure was 112/74 mmHg, pulse was irregular at 98 beats/min, and respiratory rate was at 21 beats/min. His CHA2DS2-VASc score was equal to 0, and therefore, he did not take any oral anticoagulant (OAC) agents. His physical examination was unremarkable except for a mild tenderness at the right inferior abdomen. Laboratory assessments of routine blood and urine examinations were also normal. A 12-lead electrocardiography (ECG) demonstrated an AF rhythm (see Fig. 1), chest fluoroscopy indicated dextrocardia (see Fig. 2), and abdominal fluoroscopy revealed small intestinal pneumatosis. Therefore, a working diagnosis of mesenteric arterial thromboembolism was suspected and a contrast-enhanced Computerized Tomography (CT) scan was performed. It did not reveal clear signs of mesenteric arterial thrombosis, however, did show for signs of renal and spleen infarction. Per the above findings, the final diagnosis was considered as multi-arterial thromboembolism, which probably originated from left atrium thrombosis as AF was initiated and sustained. The patient was then transferred to the Department of Cardiology and received warfarin as the OAC therapy. The international ratio (INR) was titrated to maintain a range between 2 and 3. A trans-thoracic echocardiography (TTE) showed that the left atrial diameter was 39 mm, left ventricular diameter was 48 mm, and left ventricular ejection fraction was 66%. It was also disclosed that his dextrocardia was more accurately a dextraversion than a true mirror-image. Trans-esophageal echocardiography (TEE) showed no thrombosis in the left atrium. Subsequently, radiofrequency catheter ablation of the AF was recommended. The patient agreed to receive ablation therapy and signed the informed consent forms.

**Fig. 3:**
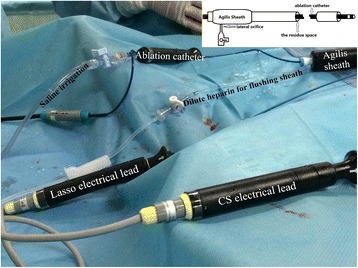
The connection of the catheters and transfusion tubes during the procedure of catheter ablation. The schematic figure showed that the inner diameter of the Agilis sheath is thicker than the ablation catheter, so there is residue space within the sheath in which thrombosis would potentially occur

Catheter ablation was performed on the fifth day following admission into the Department of Cardiology. The strategy was circumferential pulmonary vein isolation (PVI), the details of which have been previously published [3]. In brief, the irrigated ablation catheter and circumferential mapping catheter (Lasso; St. Jude Medical) were inserted into the left atrium through a steerable long sheath (Agilis sheath; St. Jude Medical) and a non-steerable long sheath (Swartz sheath; St. Jude Medical), respectively. The steerable long sheath was introduced through a right femoral vein puncture, advanced through the right femoral vein, inferior vena cava, right atrium, atrial septal puncture, and finally into the left atrium. The non-steerable long sheath was inserted via a left femoral vein puncture into the right atrium, and finally passed through the first puncture hole on the septum with guidance of a guide wire into the left atrium without a second septal puncture. Besides, a coronary sinus electrical catheter was placed into the coronary sinus ostium via a left femoral vein puncture with a 6-French short sheath. After trans-septal puncture, a single bolus of 4000 IU heparin was administered and the ACT was titrated into a range of 200 to 300 s by every 30-min testing. During the procedure, dilute heparin (500 mL per infusion bag with a final concentration of 1 IU/mL) was transfused into the steerable long sheath through its lateral orifice continuously to prevent thrombosis as there was residue space (see Fig. 3). The lateral orifice was connected to the infusion bag with an infusion set which has a normal air filter in the line and adjacent to the bag. There was also an air filter in the line of saline irrigation.

**Fig. 4:**
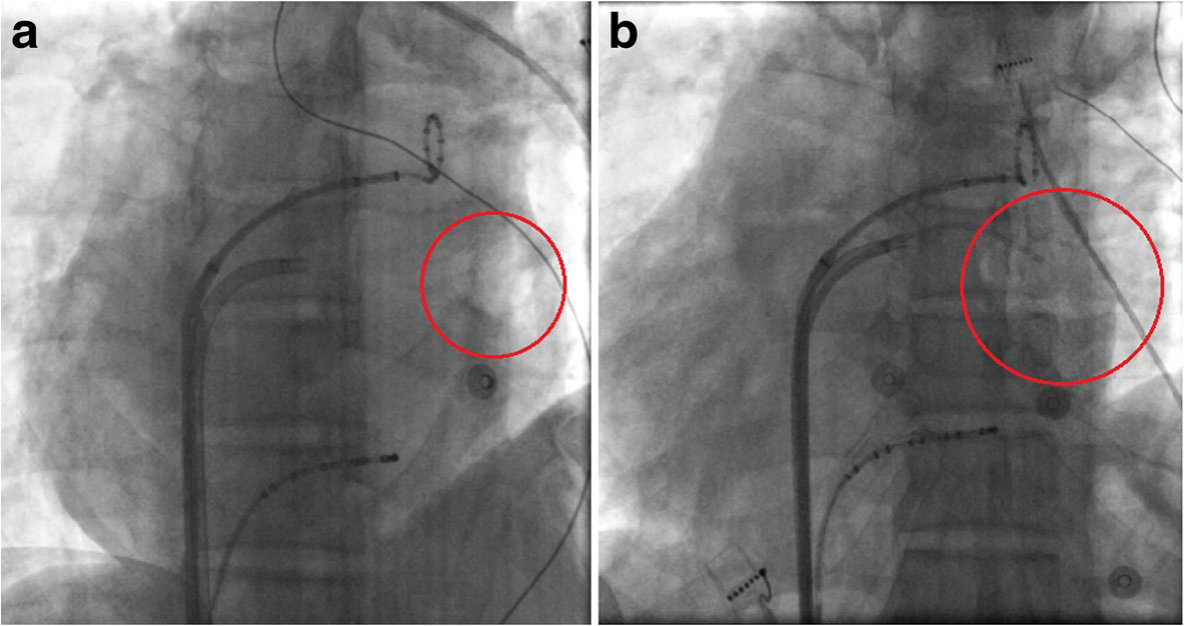
The aeroembolism accumulate in the left atrium and the process of aspiration out of it. **a**. The LAO view of the fluoroscopy demonstrated an big air bubble accumulated in the left atrium (within the red circle); **b**. A coronary angiography catheter is used to suck the aeroembolism out

The aeroembolism occurred during the process of ablation therapy when one bag of dilute heparin was depleted and the replacement of a new bag was omitted because of the operators’ negligence. It was noticed by the operator shortly, but a mass of air had already penetrated into the left atrium through the steerable long sheath and accumulated as an air bubble in the left auricle (see Fig. 4 and Additional file 1: Video 1). Although there was an air filter in the infusion line, the amount of air was so large and beyond the preventive function of the filter. Fortunately, the patient did not experience discomfort or any neurologic abnormalities because the air embolus did not occlude the systemic circulation. Meanwhile, the vital signs including blood pressure, heart and respiratory rates, and oxygen saturation were all in normal ranges. The ECG-monitor also demonstrated no ST segment of T wave changes. As compared to other cases, such a fortunate scenario would attribute to the left auricle asystole because the AF rhythm was sustained at the time it occurred, and a highest left atrium in supine position because of the dextrocardia. We then pulled the irrigated ablation catheter out of the sheath, and quickly inserted a coronary angiography (CAG) catheter into the left auricle via the steerable long sheath to remove the air embolus (see Additional file 2: Video 2). Simultaneously, we asked the patient to keep calm and hold on with the supine position as a nervous mode or a tiny move of his body might be result in a catastrophic result of arterial aeroembolism. By aspiration with a 20-mL-syringe connected to the end of the CAG catheter for several times, the air embolus was fully drawn out (see Fig. 4 and Additional file 3: Video 3). Both chest fluoroscopy and echocardiography demonstrated that no air embolus remained in the left atrium. Then, the ablation therapy was successfully completed and the AF rhythm was converted to sinus rhythm by electrical cardioversion. After discharge, monthly follow-up was performed and continued until the present time. The patient is still free of any aeroembolism events and maintains sinus rhythm.

## Discussion

Iatrogenic aeroembolism within the heart chamber during catheter ablation is a serious acute complication, but it is absolutely preventable. The incidence of vascular aeroembolism during invasive catheter insertion is reported to be, on average, less than 2% [4]. However, if such an air embolus induced an arterial aeroembolism, it could be life threatening. Morbidity and mortality of aeroembolisms are directly related to the volume of air entrainment, rate of accumulation of the air, and the position of the patient at the time it occurred [5]. A lethal volume of a vascular aeroembolism in the adult human has been estimated to be between 300 and 500 mL [6]. An upright position and negative intrathoracic pressure would increase the risk [7]. Additionally, according to this case we found that for air embolus in the left auricle, AF rhythm might be a protective factor because the asystolic left auricle would not dislodge the air embolus into arterial circulation. Early diagnosis and treatment of aeroembolism is essential and useful. Both echocardiograph and thoracic CT are available for diagnosis of aeroembolism in left atrium. Furthermore, TEE is the most sensitive method of monitoring as it can detect air emboli as small as 5 to 10 μm [8].

The goal in the treatment of aeroembolism is to prevent further air entry, reduce the volume of the air embolus, and provide haemodynamic support. The experience of this case was a heuristic lesson to teach us that paying attention to the infusion lines and air filters should be firstly executed to prevent the air emboli entry. It is also the unique and primary method to prevent serious morbidity or mortality from this complication. According to the published literature, for patients who have aeroembolism in the systemic arterial circulation, strategies such as administration of 100% oxygen or hyperbaric oxygen therapy, which could facilitate nitrogen washout and gradual reabsorption of the air, is useful to minimize the size of the air bubble [5]. In addition, anticoagulant therapy with heparin and prophylactic lidocaine administration are also recommended and potentially effective.

However, the main experience and learning point from this report is what the optimal treatment would be for a patient has an air embolus in heart chamber but has not yet advanced into arterial circulation. This rare case reminds us that the perfect method is to remove the air embolus to prevent embolism in vital organs. It was reported that using a Bunegin-Albin multiorifice catheter to aspirate the air in cases with right atrium aeroembolism could immediately improve the haemodynamic disturbance [9]. However, an easily accessible CAG catheter in a catheter lab would also be useful for the aspiration of the air embolus. With the latter, things would be more complicated and undetermined, but the following hypothetical strategies may be useful: first, with the orifice of the left auricle roughly facing the right posterior oblique direction, tilting the operating table left side up and head down would prevent the air embolus from both dislodging out of the auricle and embolizing into the cerebral circulation; second, it could be speculated that for an air embolus in left auricle that is accompanied by sinus rhythm, rapid atrial pacing may trigger AF initiation and make the left auricle temporarily asystolic; third, rapid ventricular pacing might also be partially effective because a paced ventricular tachycardia would significantly reduce the cardiac output and subsequently decrease the volume of air embolus drifting to vital organs; finally, keeping the patient calm and avoiding deep breathing might also be considered to be beneficial.

## Conclusions

In conclusion, aeroembolism during catheter ablation is a rare but life threatening complication. It requires an operator to be totally concentrated on the task with no distractions during the procedure to avoid an iatrogenic aeroembolism. An air embolus accumulated in the left auricle during AF might be associated with both a lower incidence of aeroembolism and a better prognosis than during sinus rhythm. The optimal way to address such a complication should be to aspirate the air embolus out of the heart chamber. Otherwise, the aforementioned methods might be useful to prevent deterioration.

## Additional files

Additional file 1: Video 1. Air bubble accumulated in the left atrium. (MOV 70 kb)

Additional file 2: Video 2. A coronary angiography catheter is used to suck the aeroembolism out. (MOV 522 kb)

Additional file 3: Video 3. The aeroembolism has been totally sucked out. (MOV 47 kb)

## Abbreviations

AF: Atrial fibrillation
CT: Computed tomography
ECG: Electrocardiography
INR: International ratio
OAC: Oral anticoagulation
PVI: Pulmonary vein isolation
TEE: Trans-esophageal echocardiography
TTE: Trans-thoracic echocardiography
